# Tools for efficient epistasis detection in genome-wide association study

**DOI:** 10.1186/1751-0473-6-1

**Published:** 2011-01-04

**Authors:** Xiang Zhang, Shunping Huang, Fei Zou, Wei Wang

**Affiliations:** 1 Department of Computer Science, University of North Carolina at Chapel Hill, Chapel Hill, NC, USA; 2 Department of Biostatistics, University of North Carolina at Chapel Hill, Chapel Hill, NC, USA

## Abstract

**Background:**

Genome-wide association study (GWAS) aims to find genetic factors underlying complex phenotypic traits, for which epistasis or gene-gene interaction detection is often preferred over single-locus approach. However, the computational burden has been a major hurdle to apply epistasis test in the genome-wide scale due to a large number of single nucleotide polymorphism (SNP) pairs to be tested.

**Results:**

We have developed a set of three efficient programs, FastANOVA, COE and TEAM, that support epistasis test in a variety of problem settings in GWAS. These programs utilize permutation test to properly control error rate such as family-wise error rate (FWER) and false discovery rate (FDR). They guarantee to find the optimal solutions, and significantly speed up the process of epistasis detection in GWAS.

**Conclusions:**

A web server with user interface and source codes are available at the website http://www.csbio.unc.edu/epistasis/. The source codes are also available at SourceForge http://sourceforge.net/projects/epistasis/.

## Introduction

Genome-wide association study (GWAS) examines the genetic variants across the entire genome to identify genetic factors associated with observed phenotypes. It has been shown to be a promising design to locate genetic factors causing phenotypic differences [1,2]. Since most traits of interest are complex, finding gene-gene interaction has received increasing attention in recent years [3,4]. Unlike single-locus approaches, which test and estimate the association between the phenotype and one marker (or SNP) at a time, two-locus epistasis detection approaches search for associations between the phenotype and each SNP-pair.

In general, there are two challenges in epistasis detection. The first is to develop statistical test that can effectively capture the interaction between SNPs. The second challenge is to reduce the computational burden since there are an extremely large number of SNP-pairs need to be tested in the whole genome. The computational challenge is further compounded by the multiple testing problem. Controlling family-wise error rate (FWER) and false discovery rate (FDR) are two standard approaches for controlling error rates [5]. With large number of SNPs correlated, permutation test is preferred over simple Bonferroni correction [6], which is often to conservative. The idea of permutation procedure is to randomly shuffle the phenotype values and reassign them to each sample. The test statistics of the randomly permutated data are then computed and used to estimate the distribution of test statistics under the null hypothesis. Permutation test dramatically increases the computation burden. For example, with 100,000 SNPs and 1000 permutations, the number of SNP-pairs need to be tested is about 500 billion. Efficient algorithms and software implementations are needed to enable wide applicabilities of epistasis mapping in GWAS scans.

Several approaches have been proposed for epistasis detection. For studies with a small number of SNPs, exhaustive algorithms that explicitly enumerate all possible SNP combinations have been developed [7,8]. These methods are very time consuming and cannot be applied in genome-wide studies. Heuristic approaches such as genetic algorithm [9] has also been developed. However, these approaches do not guarantee to find all significant SNP-pairs. Another common heuristic is a two-step approach [10-12]. In the first step, a subset of SNPs are selected according to certain criteria. In the second step, the selected SNPs are used for subsequent epistatic analysis. One limitation of this approach is that it misses SNPs with week marginal effects but high epistasis [13].

We have implemented a set of three two-locus epistasis detection tools that can be applied in various problem settings in GWAS. Our programs use the permutation procedure for proper error control. They are exhaustive and accurate in the sense that no significant epistatic interactions between SNP-pairs are skipped. It has been theoretically proved and experimentally validated that these programs greatly speed up the epistasis test process.

## Designing Principles

We briefly discuss the designing principles of these programs here. The detailed technical description of the algorithms behind these programs can be found in [13-15]. All the three programs utilize search space pruning to reduce the computational cost of epistatic test.

The first program is FastANOVA. It utilizes an upper bound of the two-locus ANOVA test to prune the search space. The upper bound is expressed as the sum of two terms. The first term is based on the single-SNP ANOVA test. The second term is based on the genotype of the SNP-pair and is independent of permutations. This property allows to index SNP-pairs in a 2 D array based on the genotype relationship between SNPs. Since the number of entries in the 2 D array is bounded by the number of individuals in the study, many SNP-pairs share a common entry. Moreover, it can be shown that all SNP-pairs indexed by the same entry have exactly the same upper bound. Therefore, we can compute the upper bound for a group of SNP-pairs together. Another important property is that the indexing structure only needs to be built once and can be reused for all permutated data. Utilizing the upper bound and the indexing structure, FastANOVA only needs to perform the ANOVA test on a small number of candidate SNP-pairs without the risk of missing any significant pair.

The second program COE takes the advantage of convex optimization. It can be shown that a wide range of statistical tests, such as chi-square test, likelihood ratio test (also known as G-test), and entropy-based tests are all convex functions of observed frequencies in contingency tables. Since the maximum value of a convex function is attained at the vertices of its convex domain, by constraining on the observed frequencies in the contingency tables, we can determine the domain of the convex function and get its maximum value. This maximum value is used as the upper bound on the test statistics to filter out insignificant SNP-pairs. COE is applicable to all tests that are convex.

FastANOVA and COE are designed for studies with homozygous genotypes and relatively small sample sizes. In human GWAS, the genotype is usually heterozygous, and the number of individuals can be large. We therefore developed the third program, TEAM, that is suitable for human GWAS. The basic idea of TEAM is that it incrementally updates the contingency tables of two-locus test by utilizing a minimum spanning tree. The nodes of the tree are SNPs and the edges represent the difference between two connected SNPs. It can be shown that we can get the exact test values by searching the minimum spanning tree without scanning all individuals. TEAM records the test statistics of all SNP-pairs instead of just the ones with high values. Thus it allows FWER and FDR calculation.

## Software Implementation and Overview

We provide a Web server with graphic user interface for using these tools. All three programs are implemented in C++. The source codes of both Windows and Linux versions are available for downloading.

The programs are easy to use. The inputs files include the genotype and phenotype data. The user specified parameters are the desired significance level, and the number of permutations to perform. The outputs are the significant SNP-pairs and their corresponding significance levels.

These programs are suitable for different problem settings in GWAS. FastANOVA is designed for ANOVA test that examines the association between quantitative phenotypes and binary genotypes. COE is designed for binary phenotypes and genotypes. COE supports any test statistic which is a convex function of observed frequencies in its corresponding contingency table. Both FastANOVA and COE support FWER control and are suitable for datasets with relatively small sample sizes, e.g., with less than 100 individuals. TEAM is designed for binary phenotypes but not limited to binary genotypes. It supports both FWER and FDR control. It can be applied to GWAS data with large samples(e.g. with hundreds to thousands of individuals). It can be applied to all statistical tests based on contingency tables. Detailed comparisons of the three methods can be found in Table 1.

**Table 1:** Programs and their corresponding problem settings for epistasis detection in GWAS

Program	Trait	Genotype	Error Type	Sample Size	Supported Test
FastANOVA	quantitative	binary	FWER	less than a hundred	ANOVA test

COE	binary	binary	FWER	less than a hundred	convex test

TEAM	binary	any	FWER & FDR	hundreds to thousands	test based on contingency tables

FastANOVA and COE can speed up the process of epistasis detection for about two to three orders of magnitude compared to brute force approaches. TEAM speeds up the process for about one order of magnitude, but provides wider applicability. In general, for datasets of about 100,000 SNPs and less than 100 individuals, FastANOVA and COE can be run on a single processor desktop computer, with runtime ranging from minutes to a few days depending on the parameter setting. For large human GWAS datasets, it is recommended to run TEAM on cluster. For example, for a dataset of 100,000 SNPs and 500 individuals and 100 permutations (for FDR controlling), using a 100-core cluster, the runtime is about 2 days.

## Conclusion

We provide Web server and source codes of three efficient epistasis detection tools, FastANOVA, COE, and TEAM for GWAS. These programs implement permutation procedure for proper error control and support a wide range of problem settings. They can significantly speed up the computationally intensive epistasis detection process.

## Competing interests

The authors declare that they have no competing interests.

## Authors' contributions

XZ and SH wrote the source code. All authors helped to draft the manuscript. All authors read and approved the final manuscript.
